# Conference Report: DATA ADMINISTRATION MANAGEMENT ASSOCIATION SYMPOSIUM, Gaithersburg, MD May 14–15, 1991

**DOI:** 10.6028/jres.096.041

**Published:** 1991

**Authors:** Judith Newton

**Affiliations:** Information Systems Engineering, Computer Systems Laboratory, National Institute of Standards and Technology, Gaithersburg, MD 20899

## 1. Introduction

Data administration has evolved from the need for improved planning and management of information into a resource recognized today by successful organizations as a strategic value. A primary goal of data administration is to improve the planning, organization, and management of data resources. Achieving this goal requires a clear vision for the future.

Limited resources, shifting priorities, and evolving technologies are just a few of the constraints that must be faced as data administrators attempt to translate the vision for improved data management into a reality.

The Data Administration Management Association (DAMA) is the professional organization for Data Administrators. An international board oversees a loose federation of local chapters in the United States and Australia. The National Capital Region Chapter (NCR DAMA) has monthly meetings from September through April, as well as a Symposium in May.

NCR DAMA held its fourth annual Symposium at NIST on May 14–15, 1991. The theme this year was “Managing Data—From Vision to Reality.” Attended by 225 Federal and private industry data administrators, the Symposium was cosponsored by NIST and NCR DAMA.

This year’s Symposium continued the tradition, begun with the 1990 event, of distributing the program over 2 days. However, instead of concurrent sessions, the planning committee decided on a straightforward format. In addition to the speakers, awards were presented and a report on the progress of the DAMA Working Group on Standards and Procedures was given.

## 2. Speakers

The key topics covered by the speakers included:

“Achieving the data management reality.” Arnold Barnett of Barnett Data Systems described the goals of data management, the factors which limit achievement, and the planning activities needed to accomplish the goals. One must ascertain the problems to be solved, develop a realistic plan, obtain approval to proceed, and execute the plan by building the necessary infrastructure.

“The road map for CASE implementation,” by Ian Palmer of James Martin Associates, Inc. The road map provides a variety of pathways, but generally begins with the careful selection of a pathfinder project and leads through the planned fan-out of CASE workstations backed by the necessary infrastructure development and organizational adjustments. It explicitly recognizes the cultural change implications that determine how quickly and smoothly an organization can begin to achieve the benefits of CASE and methodology.

“The impact of implementing a new information architecture.” Ron Shelby of Connecticut Mutual highlighted the role of information-driven planning, analysis, and system development techniques in organizations which use information technology to cut costs, improve client services, and react to changes in their environment. He discussed the impact of implementing an information architecture which shares data upon work process and structure.

Fred L. Forman of American Management Systems presented a fable which described what happened when data planning became an end unto itself in Dataland. Data modelers sometimes spend years without adding business value to their organization. But that could change with little or no warning. Be prepared!

“Data modeling from the data administration viewpoint,” by Joseph H. Oates of Life Cycle Technology. A data sharing environment raises many issues that have not been a concern to application developers in the past. Data administrators follow a much more rigorous set of rules in developing a global data model than is required for the data models of systems which do not share data.

“Data management and information proficiency: a vision for the future,” by Thomas J. Buckholtz of the General Services Administration. Information proficiency is the effective use of information to accomplish an individual’s job and an agency’s mission. Essential to this process is the successful management of data to maximize accessibility, accuracy, consistency, relevance, and strategic use. Vision, technology, people, organizations, and tools are critical elements in the equation of effective data management.

“Information reengineering,” presented by William R. Durell of Data Administration, Inc. Unfortunately, most data reengineering efforts result in minor or cosmetic improvements to data quality. Cosmetic, moderate, and reconstructive (severe) methods may be applied by resorting to a variety of strategies. A set of risks and benefits attends each method.

Graham W. Thompson of Manager Software Products discussed “Managing information across multiple CASE tools.” The open tool architecture concept within AD/Cycle calls for the sharing of information via the repository between your organization’s tools of today and tomorrow. He addressed the practicality of this vision and the steps that individual organizations and the industry as a whole must take to make this concept a reality.

“Implementing data architecture,” by William H. Inmon. Five years ago, data architecture was an interesting idea. Three years ago, there were perhaps five companies in the process of implementing an architected approach to data. Today, there are many companies either in the process of completing their first thrusts into data architecture or actually using their first implementation. Some success stories and lessons learned along the way were presented.

“Data Management and customer satisfaction.” Karen Lindsay of Blue Cross and Blue Shield of Georgia discussed the importance of customer satisfaction in the corporate culture. Business success can be achieved through refocusing on working with customers versus around them. Data professionals must monitor performance and continually be working to improve the process which will allow data administration and customers to achieve long-term goals and avoid short-term frustrations.

“Data administration, the IBM repository, and CASE technology at Depository Trust Company.” Emmanuel Ackerman presented his views on the state of the IBM repository in the short and long term, the DTC’s experience working with it, and the steps being taken to position for future releases. The most important step has been an effort to implement an application development methodology and Knowledgeware’s Information Engineering Workbench (IEW/ADW).

“Status and application of standards for data administration,” Bruce K. Rosen, Computer Systems Laboratory, NIST. There are many data processing related standards in the world today. Sorting through these standards and applying them is often a difficult task, complicated by a lack of knowledge about the current status of different standards. Identification and proper application of data administration related standards is essential.

“The need for future vision in a data management program.” Reed Phillips of the Department of Commerce presented examples of future vision in the public and private sectors as representative of global vision statements that are indicative of problems and issues in their respective communities. He discussed data management as it relates to major Department of Commerce missions, together with vision statements that reflect individual bureau and department-wide perspectives.

## 3. Working Group Report

The Working Group on Standards and Procedures has been meeting monthly during the past year. It has produced a draft Model Data Administration Standards Manual, which was distributed for comment. This document, when in final form, will be circulated as a guide to the administration of information standards and the procedures for implementation. The group plans to resume monthly meetings in the fall.

## 4. Proceedings

The proceedings of this Symposium will be released as a NIST publication. The Proceedings of the First^1^ and Second^2^ Annual DAMA Symposia were published by NIST and copies are still available. The Fifth Annual Symposium will be held at NIST May 12–13, 1992.

## Footnotes

1 Judith J. Newton and Frankie E. Spielman, eds., Data Administration: Management and Practice, Proceedings of the First DAMA Symposium, NIST Special Publication 500–159, National Institute of Standards and Technology, Gaithersburg, MD, October 1988.

2 Judith J. Newton and Frankie E. Spielman, eds., Data Administration: Standards and Techniques, Proceedings of the Second Annual DAMA Symposium, NISTIR 90-4292, National Institute of Standards and Technology, Gaithersburg, MD, April 1990.

